# PhyloNext: a pipeline for phylogenetic diversity analysis of GBIF-mediated data

**DOI:** 10.1186/s12862-024-02256-9

**Published:** 2024-06-11

**Authors:** Vladimir Mikryukov, Kessy Abarenkov, Shawn Laffan, Tim Robertson, Emily Jane McTavish, Thomas Stjernegaard Jeppesen, John Waller, Matthew Blissett, Urmas Kõljalg, Joseph T. Miller

**Affiliations:** 1 Institute of Ecology and Earth Sciences, University of Tartu, Liivi 2, Tartu, 50409 Estonia; 2 Natural History Museum, University of Tartu, Vanemuise 46, Tartu, 51003 Estonia; 3 Earth and Sustainability Science Research Centre, School of Biological, Earth and Environmental Sciences, University of New South Wales, Sydney, Australia; 4 Global Biodiversity Information Facility, Universitetsparken 15, Copenhagen, 2100 Denmark; 5 School of Natural Sciences, University of California, Merced, USA

**Keywords:** Big data, Biodiversity, Data cleaning, Derived datasets, GBIF, Open Tree of Life, Phylodiversity, Spatial phylogenetics

## Abstract

**Background:**

Understanding biodiversity patterns is a central topic in biogeography and ecology, and it is essential for conservation planning and policy development. Diversity estimates that consider the evolutionary relationships among species, such as phylogenetic diversity and phylogenetic endemicity indices, provide valuable insights into the functional diversity and evolutionary uniqueness of biological communities. These estimates are crucial for informed decision-making and effective global biodiversity management. However, the current methodologies used to generate these metrics encounter challenges in terms of efficiency, accuracy, and data integration.

**Results:**

We introduce PhyloNext, a flexible and data-intensive computational pipeline designed for phylogenetic diversity and endemicity analysis. The pipeline integrates GBIF occurrence data and OpenTree phylogenies with the Biodiverse software. PhyloNext is free, open-source, and provided as Docker and Singularity containers for effortless setup. To enhance user accessibility, a user-friendly, web-based graphical user interface has been developed, facilitating easy and efficient navigation for exploring and executing the pipeline. PhyloNext streamlines the process of conducting phylogenetic diversity analyses, improving efficiency, accuracy, and reproducibility. The automated workflow allows for periodic reanalysis using updated input data, ensuring that conservation strategies remain relevant and informed by the latest available data.

**Conclusions:**

PhyloNext provides researchers, conservationists, and policymakers with a powerful tool to facilitate a broader understanding of biodiversity patterns, supporting more effective conservation planning and policy development. This new pipeline simplifies the creation of reproducible and easily updatable phylogenetic diversity analyses. Additionally, it promotes increased interoperability and integration with other biodiversity databases and analytical tools.

## Background

Phylogenetic diversity metrics, such as the expected loss of phylogenetic diversity and the evolutionarily distinctness and global endangerment (EDGE) score, have been recommended as indicators for tracking progress towards the goals of the Global Biodiversity Framework established at COP15 in Montreal in December of 2022 to protect and manage biodiversity. However, the limited availability of curated spatial and phylogenetic data, coupled with the challenges in automating the connection between these data, poses significant challenges in providing accurate phylogenetic diversity indicators. Another constraint in the application of spatial phylogenetics is the necessity for users to master multiple complex software tools to analyze data. This requirement often exceeds the technical expertise typically possessed by researchers and policy teams, impeding reproducible science.

Over time, there has been a notable explosion in the availability of biodiversity data, largely attributable to the development of global infrastructures and projects such as the Global Biodiversity Information Facility (GBIF; https://www.gbif.org/), Open Tree of Life (OpenTree; https://tree.opentreeoflife.org/), and the newly developed ChecklistBank, a collaborative initiative between GBIF and the Catalogue of Life (COL; https://www.catalogueoflife.org/). These platforms have significantly expanded both in size and taxonomic breadth, offering researchers a diverse array of tools to efficiently filter and utilize data to meet their specific research needs. With more than 2.93 billion worldwide species occurrence records (as of April 2024), GBIF data is widely utilized as input data for species distribution modeling and other scientific disciplines [1]. Meanwhile, OpenTree aims to construct a comprehensive, dynamic, and digitally accessible tree of life by combining published phylogenetic trees with taxonomic data. Currently, the Open Tree of Life (OToL) phylogeny contains the backbone of all eukaryotic life on earth and contains over 2.3 million terminals – generally species, with 112,669 terminals supported by phylogenetic data from 1,331 published studies (https://tree.opentreeoflife.org/about/synthesis-release/v14.9). The integration of user-curated data input tools in OpenTree, coupled with the taxonomic crosswalk between GBIF and OpenTree identifiers [2], presents researchers with enhanced capabilities for expert analysis. Furthermore, the advent of ChecklistBank serves to streamline and improve the critical process of name matching, thereby augmenting the efficiency and precision of taxonomic alignments.

Traditionally, diversity measures such as the Simpson index or the Shannon index have used the number of species (species richness) and their abundances to compare diversity between two areas. However, this approach ignores phylogenetic diversity: protecting a large number of evolutionarily distinct organisms is likely to preserve more evolutionary information than protecting many closely related organisms [3]. Spatial phylogenetics has emerged as a burgeoning field that uses diversity indices, coupled with spatial and phylogenetic data [4, 5], to measure biological communities in terms of their evolutionary histories. The application of these indices can assist in the conservation of ecological, genetic, or functional diversity. However, the process of developing a phylogeny, curating spatial data, name matching, and conducting spatial phylogenetic analyses is both time-consuming and labor-intensive. This complexity requires specialized expertise and resources, particularly the ability to develop phylogenies from large genetic databases and map these phylogenies to biodiversity data sources such as GBIF, which in turn limits the use of GBIF data for purposes beyond research, such as informing policy decisions. Consequently, global-scale species-level analyses for major lineages have not been attempted, and conducting such analyses at a national scale is often unrealistic [5].

To address these issues, we have developed a flexible pipeline, PhyloNext, which integrates the standard analytical steps required for phylogenetic diversity analysis. PhyloNext encompasses data curation and analysis pipelines for evaluating phylogenetic diversity, directly using GBIF occurrence data and a synthetic phylogeny from OpenTree as input data. Emphasizing modularity, efficiency, and automation in data preparation, we designed the pipeline to streamline the data preparation and analysis workflow. This integration of well-established methods into a cohesive and user-friendly system will significantly reduce the time and effort required by researchers. Consequently, PhyloNext has emerged as a valuable tool for both the research and policymaking communities, particularly benefiting those who find the technical aspects of data preparation challenging. To validate the efficacy of our approach, we compare the results of our analyses with those obtained from a previous study that utilized a carefully curated occurrence dataset and a well-studied phylogeny.

## Implementation

### Overview

The PhyloNext pipeline was developed to efficiently integrate GBIF occurrence data and OpenTree phylogenies as they pass through data analysis using Biodiverse software [4]. Designed to streamline the process and maximize accuracy, the pipeline is suitable for local use or on any cloud-based or high-performance cluster-based platforms. The pipeline minimizes laborious, potentially error-prone manual steps and generates intermediate result files at each step to provide transparency. The PhyloNext pipeline emphasizes flexibility and user control by allowing users to adjust the default settings to meet their specific requirements at each step, including selecting the geographic, taxonomic, or temporal scope of analysis and determining the inclusion or exclusion of GBIF occurrences that may be putative outliers.

The PhyloNext pipeline involves several steps:

1. Filtering of GBIF species occurrences for specified taxonomic groups or clades and geographic areas;
2. Data filtering (e.g., removal of spatial outliers and unreliable records);
3. Spatial binning of species occurrences (to aggregate data into manageable units and reduce spatial noise);
4. Preparation of phylogenetic tree and species name-matching with GBIF species IDs;
5. Estimation of phylodiversity indices using Biodiverse;
6. Export and visualization of the obtained results.

**Fig. 1:**
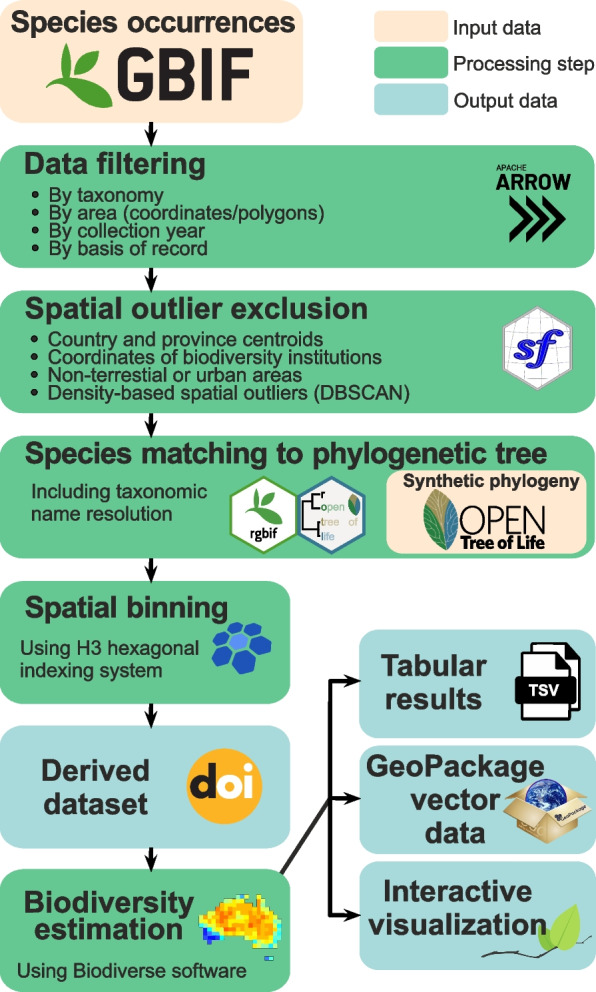
An overview of general workflow with PhyloNext

As the PhyloNext pipeline performs multiple processes (Fig. 1), it was built using the Nextflow workflow manager [6]. Nextflow is designed to help users run complex pipelines of processes, potentially involving multiple steps and dependencies between them, in an efficient and reproducible manner. One of the key features of such tools is the ability to run processes in parallel, which can help to reduce the overall time taken to complete a workflow by distributing the workload across multiple computational resources. To run the pipeline effectively and maximize resource utilization, Nextflow uses a scheduling algorithm to determine the order in which processes should be run based on the dependencies between them and the availability of resources, and effectively and automatically allocates CPUs on the available computational resources (whether a local computer, a cluster, or in a cloud environment).

The core steps of the pipeline are implemented in R [7], Python [8], and Perl [9] languages. To handle larger-than-memory datasets, PhyloNext relies on the *arrow* [10] R package as an interface to the Apache Arrow C++ library. For fast operation on large tables and data aggregation we use the *data.table* package [11]. Species name matching is performed using *rgbif* [12] and *rotl* [13] packages. Spatial binning is done using Uber’s H3 hexagonal hierarchical geospatial indexing system (https://h3geo.org/) and performed with *h3* package [14]. Processing of spatial data is performed using the *sf* package [15]. Diversity estimation is done using Biodiverse v.4 [4]. The *leaflet* package [16] is used for interactive visualization of the results. All software dependencies required for PhyloNext were packaged into a set of Docker and Singularity/Apptainer [17] containers providing a more straightforward setup and workflow reproducibility than having to install the packages and tools individually, which ensures the consistency of results.

The main output of the pipeline comprises diversity estimates in tabular format and interactive map visualizations. To enable platform independence and portability of the results, geospatial information (H3 polygons) along with the diversity estimates are also exported using the GeoPackage format, which is supported in most modern GIS software packages (e.g., QGIS). In addition to the quality-filtered data, PhyloNext creates a list of sources used in the analysis to properly credit GBIF data providers and Open Tree of Life phylogenetic data contributors. Using this list for species occurrences, a derived dataset can be registered and a persistent identifier (a citable record with a unique DOI) obtained through the GBIF service (https://www.gbif.org/derived-dataset).

We provide extensive documentation and tutorials on the pipeline website (https://phylonext.github.io/).

### Data filtering and cleaning

Data filtering and cleaning constitute key steps in ecological research, serving to customize the dataset in alignment with specific research needs and objectives. To narrow down the diversity analysis, the PhyloNext pipeline facilitates the specification of taxonomic, spatial, and temporal scopes.

The *taxonomic scope* can be defined by selecting a taxon or a set of taxa from the Linnaean hierarchy using phylum to genus ranks (multiple comma-separated values allowed in PhyloNext; e.g., command-line option `--family "Felidae,Canidae"`) or providing a list of numerical species keys based on the GBIF Backbone Taxonomy [18] (`--specieskey` option).

The *spatial scope* can relate to geographical regions defined by a coordinate box (minimum and maximum latitude and longitude values; e.g., `--lonmin 47.0 --lonmax 55.5`), country codes (following ISO 3166 standard; multiple comma-separated values allowed; e.g., `--country "DE,PL,CZ"`), or polygons based on the World Geographical Scheme for Recording Plant Distributions (WGSRPD [19]) covering the whole world with units of different spatial levels (continental or regional; e.g., `--regions "L1_AFRICA").

The *temporal scope* is defined by a temporal window, which sets the upper and lower bounds of the sample collection date (e.g., `--minyear 1950 --maxyear 2000`). In the analysis of diversity dynamics, this option allows for flexibility in choosing the optimal temporal data aggregation, dependent on the life history and population ecology properties of the studied taxon [20]. The collection date can also be utilized in more nuanced temporal analyses. For instance, using a sliding temporal window can capture transient yet significant changes in biodiversity. Moreover, including only those species present in the initial temporal window for subsequent analyses (e.g., using the `--specieskey` option) can help to exclude the impact of invasive species on phylogenetic diversity trends within native biota.

#### Occurrence filtering, outlier removal, and binning

To remove occurrences that are potentially unreliable or non-target, PhyloNext allows users to filter data by GBIF data fields such as collection date (minimum year of species occurrence), the basis of record, coordinate precision and uncertainty. For example, filtering based on the “basis of record” field can exclude extinct species and observations (data originating from citizen science projects such as iNaturalist; e.g., `--basisofrecordexclude "FOSSIL_SPECIMEN,HUMAN_OBSERVATION"`), or it can be used to focus only on preserved specimens (those preserved in herbaria or zoological collections; e.g., `--basisofrecordinclude "PRESERVED_SPECIMEN"`). Filtering based on coordinate precision and uncertainty can be applied to remove records with imprecise or rounded coordinates (e.g., `--coordprecision 0.1`). Collection date can also be used to remove occurrences with low geographical accuracy recorded in the pre-GPS era or to focus only on the most recent data.

PhyloNext supports several options to improve the accuracy of species occurrence patterns and reduce biases in diversity estimates, including filtering by GBIF data quality flags and incorporating separate data cleaning algorithms within the workflow. One method to remove common spatial errors in species occurrence data involves identifying erroneous coordinates that are assigned to country and province centroids or country capitals, often resulting from automated geo-referencing based on ambiguous locality descriptions [21]. Additionally, records of cultivated or captured individuals located in botanical gardens and zoos, as well as samples with coordinates corresponding to museums, can be excluded using the biological institution filter. Non-terrestrial species occurrences (e.g., records with swapped latitude and longitude that appear in oceans or lakes) and occurrences from urban areas can also be identified and removed by cross-referencing record coordinates with corresponding polygon data. For these tasks, PhyloNext currently utilizes shapefiles from the Natural Earth project (https://www.naturalearthdata.com/).

Density-based clustering (DBSCAN) can be used to identify and remove spatial outliers in species occurrence data by grouping points that are close to each other based on their spatial coordinates. Points that are far from any dense clusters can be considered as putative outliers, such as occurrences of a species outside its natural area or instances of species misidentification, and thus could be removed. However, it is crucial to recognize that sparse species distributions do not inherently indicate inaccuracies. Developing a method to accurately determine the natural distribution of a species would greatly enhance this process. The PhyloNext pipeline, with its modular structure, is designed to accommodate such advancements. In the future, once a reliable method becomes available, it could be seamlessly integrated into PhyloNext, enabling more effective and robust data filtering.

After data filtering and outlier removal, PhyloNext performs spatial binning of species occurrences using the Discrete Global Grid System (DGGS). This system discretizes the Earth's surface into a tessellated array of regular, geo-referenced, and indexed cells. This approach significantly enhances the management of large datasets by eliminating the need for cartographic projection transformations, which are not only computationally intensive but also introduce artifacts and uncertainties. Unlike traditional Cartesian grid cells, DGGS accounts for the Earth's curvature, providing cells of nearly uniform size and shape across all surfaces of the Earth and consistent spatial resolution everywhere. In the PhyloNext pipeline, we specifically utilize the Hexagonal Hierarchical Geospatial Indexing System (H3; https://h3geo.org/), an open-source tool developed by Uber. The H3 library is compatible with all major programming languages, offering a robust programming interface and supporting multiple resolutions, from very coarse (resolution 0) to extremely detailed (resolution 15, with cells less than 1 m2). For global-scale diversity analysis, resolutions 3 and 4 are most effective, providing an average cell area of 12,393 km2 (edge length 69 km) and 1770 km2 (26 km), respectively. In this hierarchical system, each cell has a unique index, such as `8a1f05835a37fff` for GBIF headquarters, facilitating efficient data organization and retrieval.

### Phylogenetic trees

PhyloNext provides two distinct methods for including phylogenetic trees in the analysis. Firstly, users can supply a pre-constructed phylogenetic tree as input in either Newick or Nexus formats. Secondly, a tree can be obtained through automatic retrieval from OpenTree using its dated phylogeny API (https://github.com/OpenTreeOfLife/chronosynth). Open Tree provides a comprehensive synthetic phylogenetic tree synthesized based on sets of published phylogenetic trees augmented with taxonomic data [22]. Open Tree utilizes taxonomic data to augment the structure and completeness of the synthetic tree when source phylogenies are absent or sparsely sampled. The dating algorithm applies published date estimates to internal nodes in the synthetic tree, and uses those dates to infer approximate dates for nodes where no date estimates are available using *bladj* as part of the *phylocom* package [23]. Users can also choose to remove branches lacking phylogenetic support from the tree by utilizing the `*--phylo_only*` argument. To correlate between phylogeny and occurrence datasets, the OpenTree taxonomy is matched to GBIF taxonomy using the taxonomic name resolution service (TNRS) of OToL [2]. For the synthetic tree retrieved from OToL, the output also includes a list of citations for the studies where the original phylogenetic trees were published, ensuring proper attribution and credit.

Depending on the source of a user-provided phylogenetic tree, PhyloNext offers two options for the format of tip labels. Users can choose between Latin binomials and OpenTree IDs (OTT) as tip labels. To align Latin binomials with the GBIF backbone taxonomy, the TNRS of GBIF [24] is used. For OpenTree IDs, users must specify the particular taxonomy group within the Open Tree of Life (by default, this is set to "All life") and species name matching is then conducted using the TNRS of OToL [2].

### Biodiversity estimation and visualization

Numerous metrics have been developed in the biodiversity estimation field that provides valuable information on various diversity and endemicity aspects. A distinct category of these metrics considers the phylogenetic relationship among species. Such metrics are essential for understanding the composition of complex biological communities, their evolutionary history, and their potential response to environmental or anthropogenic disturbances.

Biodiverse [4] is a program that supports the most comprehensive list of spatial phylogenetic indices. The most widely used indices, which form the baseline for the PhyloNext pipeline, are listed in Table 1. Biodiverse calculates metrics that measure the diversity and uniqueness of species in a dataset as well as performs estimation of standardized effect sizes (SES; Z-scores). These Z-scores measure the deviation of the observed community structure from that expected under a null model of random species distribution. The null model is obtained by estimating the diversity index of interest by simulating multiple random communities.

**Table 1:** The most commonly used diversity and endemicity indices and their interpretation

Metric	Description	Reference
Species richness (R) or taxonomic diversity (TD)	The number of unique species found at the location (species have identical contribution independent of their evolutionary relationships)	-
Phylogenetic diversity (PD)	Phylogenetic equivalent of species richness, taking evolutionary divergence into account	[25]
Relative phylogenetic diversity (RPD)	Over-representation of long or short branches in the community	[26]
Range-weighted endemism (WE) and its corrected form (CWE)	Concentration of range-restricted species	[27]
Phylogenetic endemism (PE) and its corrected form	Spatial rarity of PD (the amount of PD entirely restricted to a given area)	[28]
Relative phylogenetic endemism (RPE)	Over-representation of rare (lineages with restricted range sizes) long or short branches in the community	[26]
Categorical Analysis of Neo- and Paleoendemism (CANAPE)	Quantifies areas of neo- and paleo-endemism	[26]

The `*rand_structured*` randomization algorithm is the default in Biodiverse. It shuffles species occurrences randomly across the entire study area while preserving the species richness of each sample or grid cell. This method is effective for most studies. However, for large-scale analyses encompassing different biomes or continents, where the total pool of species may span multiple environments, this approach might not be ideal. For instance, such randomizations may allocate a polar taxon to the tropics, or a desert taxon to a rainforest. To mitigate this issue, Biodiverse supports randomizations within a subset of data, based on spatial conditions such as biomes or zoogeographic regions. Users of PhyloNext can specify these conditions using a GeoPackage file containing multiple polygons. This feature ensures that species remain within their native biome while still being randomly relocated, thus allowing the randomization process to be more suitably tailored for the specific needs of the study and ensuring that the results accurately reflect the distribution of species in the area of interest.

In general, SES can be computed for various diversity metrics. However, the estimation of SES represents one of the most computationally intensive steps in the workflow. To increase the processing speed, PhyloNext enables splitting of randomization tasks into chunks, which can then be run in parallel.

Nearly all diversity estimates depend on sampling effort. To address this, a redundancy index [5] is used to identify potential bias in undersampled grid cells. Defined as 1 - (species richness / number of records), the index quantitatively assesses cells with inadequately sampled species communities. This facilitates identification and the exclusion or adjustment of cells with insufficient data in analyses. In statistical models, the redundancy index can serve as an offset variable, accommodating the varying degrees of sample completeness across cells and thus mitigating sampling bias. Furthermore, the index can act as a weighting factor in analyses, prioritizing observations from cells with more reliable data.

To enhance the interpretability of the pipeline's results, the biodiversity estimates are mapped using the Leaflet library, generating an interactive HTML file that can be visualized through a web browser. Users can zoom in/out and select which indices to display on the map. Additionally, the exact biodiversity values are displayed by hovering over specific grid cells, providing an efficient way to explore and analyze the results. Maps from the open-source, volunteer-maintained OpenStreetMap (OSM; https://www.openstreetmap.org) project are used as a basemap layer.

The biodiversity estimations, along with the phylogenetic tree, are saved as native Biodiverse format files (BaseData, or BDS, file). These files can be loaded using the Biodiverse GUI (https://shawnlaffan.github.io/biodiverse/) to explore and visualize the distribution of specific phylogenetic lineages across the grid cells of a map.

**Fig. 2:**
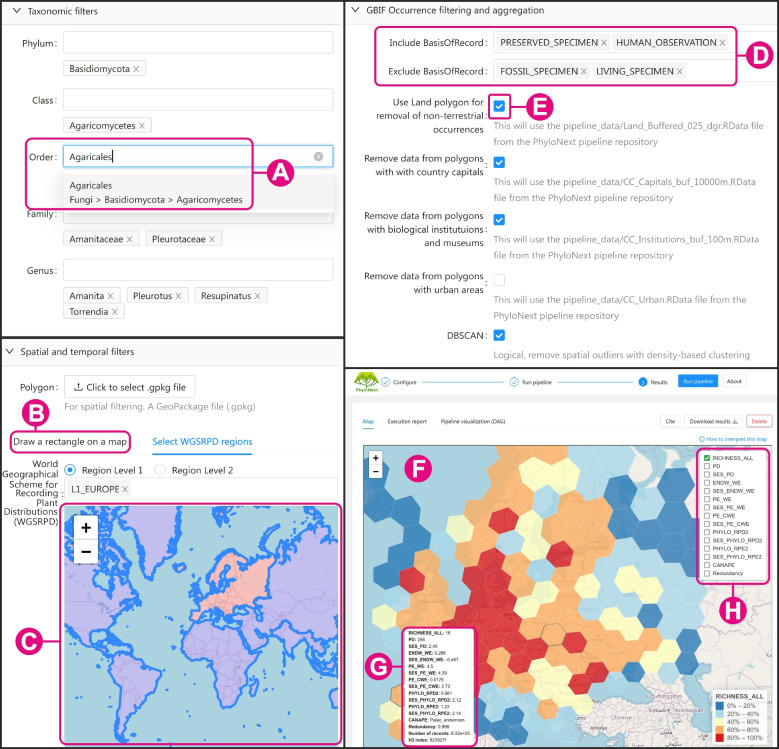
A screenshot of the PhyloNext web-GUI (only several fragments of the GUI are shown). **A** Dropdown menu for autocompleting taxon names. **B** Option to select a spatial region of interest by manually drawing a rectangle on the map. **C** Point-and-click selection of the WGSRPD regions. **D** Inclusion and exclusion of different classes of species occurrences based on the basis of record type. **E** Features for removing various types of spatial outliers. **F** Interactive, zoomable map displaying analysis results. **G** Popup window displaying diversity estimates for a grid cell when the mouse pointer is hovered over it. **H** Panel for selecting the diversity index to display on the map

A user-friendly, web-based graphical user interface (GUI) has been developed to facilitate easy and efficient navigation for exploring the pipeline (Fig. 2). This interface offers a simplified approach to accessing phylogenetic diversity information, requiring minimal technical expertise. Built as a single-page application using the React.js JavaScript framework, the GUI effectively controls the PhyloNext pipeline by communicating with a REST web service written in Node.js. To access the GUI, users must have a GBIF user account and can visit https://phylonext.gbif.org/. The source code for the GUI can be found at https://github.com/gbif/phylonext-ui/, while the web service code is available at https://github.com/gbif/phylonext-ws.

### Pipeline usage

The typical command for running the pipeline is as follows:

nextflow run vmikk/phylonext -r main \

 --input "/mnt/GBIF/Parquet/2024-01-01/" \

 --genus "Acacia" \

 --country "AU" --minyear 1950 \

 --phytree "Acacia.nwk" \

 --dbscan true \

 --iterations 1000 \

 -profile docker

This will launch the pipeline with the `docker` configuration profile and perform the analysis of phylogenetic diversity and endemism of *Acacia* species communities observed in Australia since 1950. In addition to removing records with common geospatial issues, DBSCAN-based removal of outliers will be performed.

The PhyloNext pipeline generates a comprehensive and easily accessible output, including an interactive map displaying the diversity values for rapid result visualization. All diversity estimates are available in tabular format and GeoPackage format, compatible with most modern GIS software, facilitating integration into further analyses, such as investigating environmental or anthropogenic drivers of diversity. However, prior conducting further analyses, it is crucial to ensure harmonization and alignment between the diversity and explanatory datasets. The latter may be formatted as raster pixels, square grids, or irregular polygons and may not align neatly not only with H3 polygons of the PhyloNext output but also between each other. Dataset harmonization often requires resampling or interpolating data to a common resolution, or spatially averaging raster values.

Additionally, the pipeline produces a filtered dataset that includes species occurrences, which, like the diversity outputs, is convenient for further analyses or modeling. In conjunction with this dataset, PhyloNext provides a file containing references to the primary sources of the data and Digital Object Identifiers (DOIs) associated with these datasets. These DOIs can be employed for citing the data and giving proper credit to the original data providers who collected and curated the data. This file also enables the creation of a derived dataset, which is a citable entity in itself, possessing a unique DOI (https://www.gbif.org/derived-dataset/about).

## Results

**Fig. 3:**
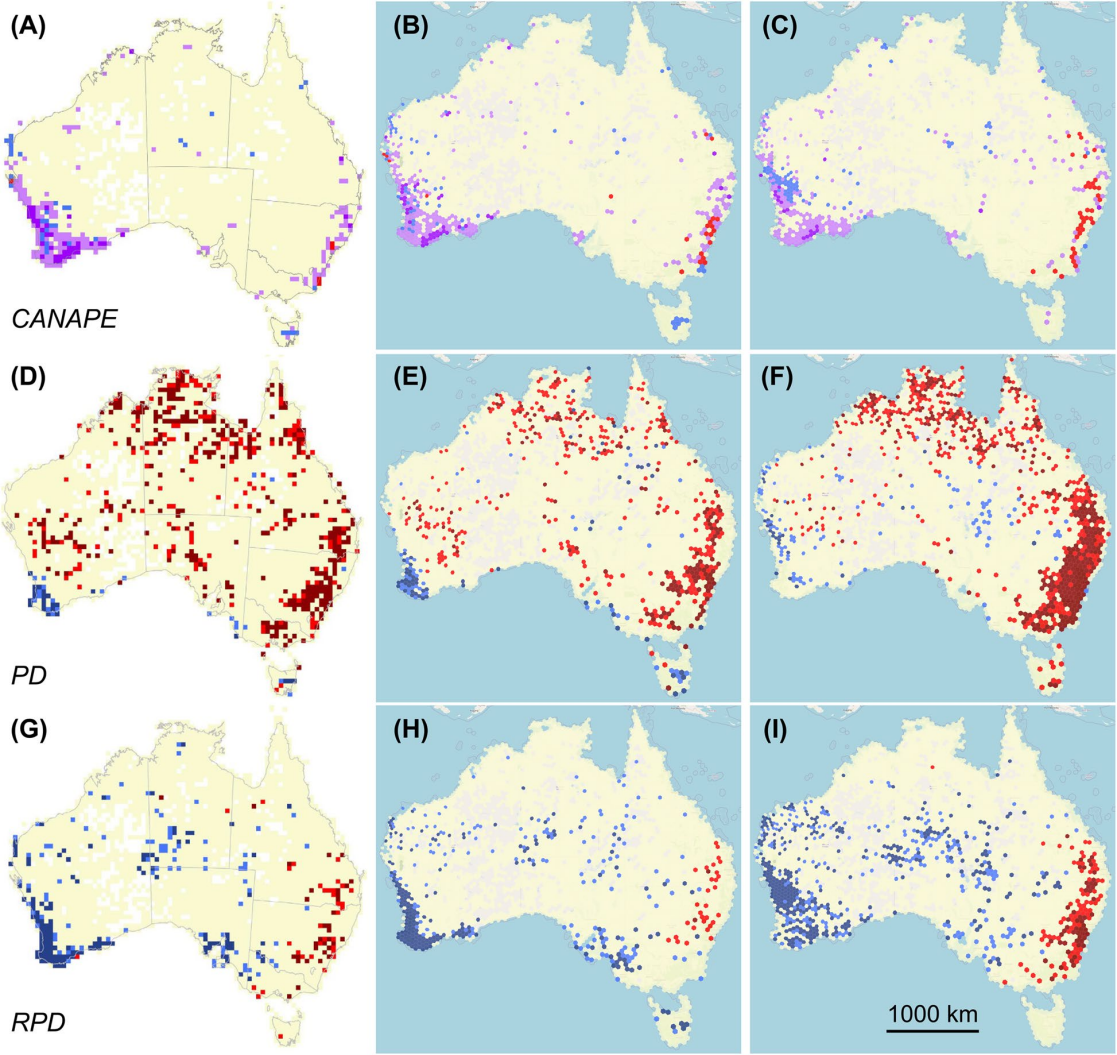
Comparison of phylogenetic diversity metric of the original study (left column [26]) with the PhyloNext results obtained using a benchmark dataset (middle column) and dataset with species occurrences derived from GBIF and phylogenetic tree from OToL (right column). **A-C**, the red values indicate grid cells that contain centers of neo-endemism; blue, paleo-endemism; light purple, a mixture of neo-endemism and paleo-endemism; dark purple, centers of super-endemism. **D-I**, red values indicate grid cells that contain significantly less PD or RPD than expected; blue, significantly more PD or RPD than expected. Beige cells are not significant, cells without color contain no records. Images from the left column are republished from [26] with permission

To demonstrate the capabilities of the PhyloNext pipeline, we benchmarked it against published studies [26, 29, 30] (Fig. 3) that used a well-curated dataset of the Australian plant genus *Acacia*. To make the original dataset compatible with PhyloNext, it was converted into the Apache Parquet file format. In the original dataset, species occurrences were in the Lambert Conformal Conic projection for Australia, which were converted to WGS 84 latitude and longitude coordinates (EPSG:4326). In addition, several of the 508 *Acacia* species in the study were renamed according to the current classification system. To restrict analysis to the same species as in the original dataset, we subsetted GBIF occurrence data by the GBIF species keys from the original dataset and selected records marked as preserved specimens in the basis of record field.

We used the custom synthesis option in OpenTree [31] to build a synthetic phylogeny of *Acacia*. Phylogeny was generated from four input trees, which included the original input tree [26] as well as the other auxiliary trees [32–34]. Only tree tips with phylogenetic support were considered, tips imputed based on taxonomic data only were excluded. Clade ages from [35] were used to generate a smoothed chronogram via the OpenTree dated phylogeny API (https://github.com/OpenTreeOfLife/chronosynth).

We conducted a comprehensive test involving four distinct analyses to assess the impact of different datasets on the diversity estimates. The test included: a) a nearly identical analysis of the original spatial dataset and phylogeny, b) analysis of original phylogeny with new filtered GBIF occurrence data with the original phylogeny, c) analysis of the original occurrence data with a custom synthetic phylogeny from OpenTree, and d) analysis of new filtered GBIF occurrence data with a custom synthetic phylogeny from OpenTree. In these analyses, we generated a consistent set of Biodiverse-derived metrics and visualizations (Table 2). To obtain standardized effect sizes of diversity measures, we performed 5000 spatially-unconstrained randomization iterations. All PhyloNext commands utilized in this study are available in the GitHub repository at https://github.com/vmikk/PhyloNext_manuscript.

**Table 2:** Concordance of diversity estimates obtained using the PhyloNext pipeline for the benchmark dataset and the dataset based on GBIF- and OToL-derived data

Diversity index	Concordance of estimates
*Correlation (continuous variables)*	
Species richness	*r* = 0.90, *p* << 0.05
PD	*r* = 0.83, *p* << 0.05
PE WE (phylogenetic endemism)	*r* = 0.87, *p* << 0.05
RPD	*r* = 0.68, *p* << 0.05
*Agreement (nominal variables)*	
CANAPE^a^	0.85 (*n* = 1904)
CANAPE^b^	0.57 (*n* = 364)
SES PD^a^	0.63 (*n* = 1904)
SES PD^b^	0.63 (*n* = 721)

^a^Rand index across all grid cells

^b^Rand index across grid cells with at least one cell in a pair is statistically significant

PhyloNext was successful in reproducing the original analysis, as evidenced by the similar pattern observed across all metrics and maps (Fig. 3). However, due to the slight data transformation, changes in grid scale, and stochasticity of data randomization during effect size estimation, it is reasonable to expect some variation between the original and replicated maps. Notably, areas of super-endemism are observed in both the southwestern and southeastern regions of Australia. Different combinations of the two phylogenies and two datasets also yielded consistent results (Fig. 3 and Table 2) indicating the pipeline and metrics are robust.

**Fig. 4:**
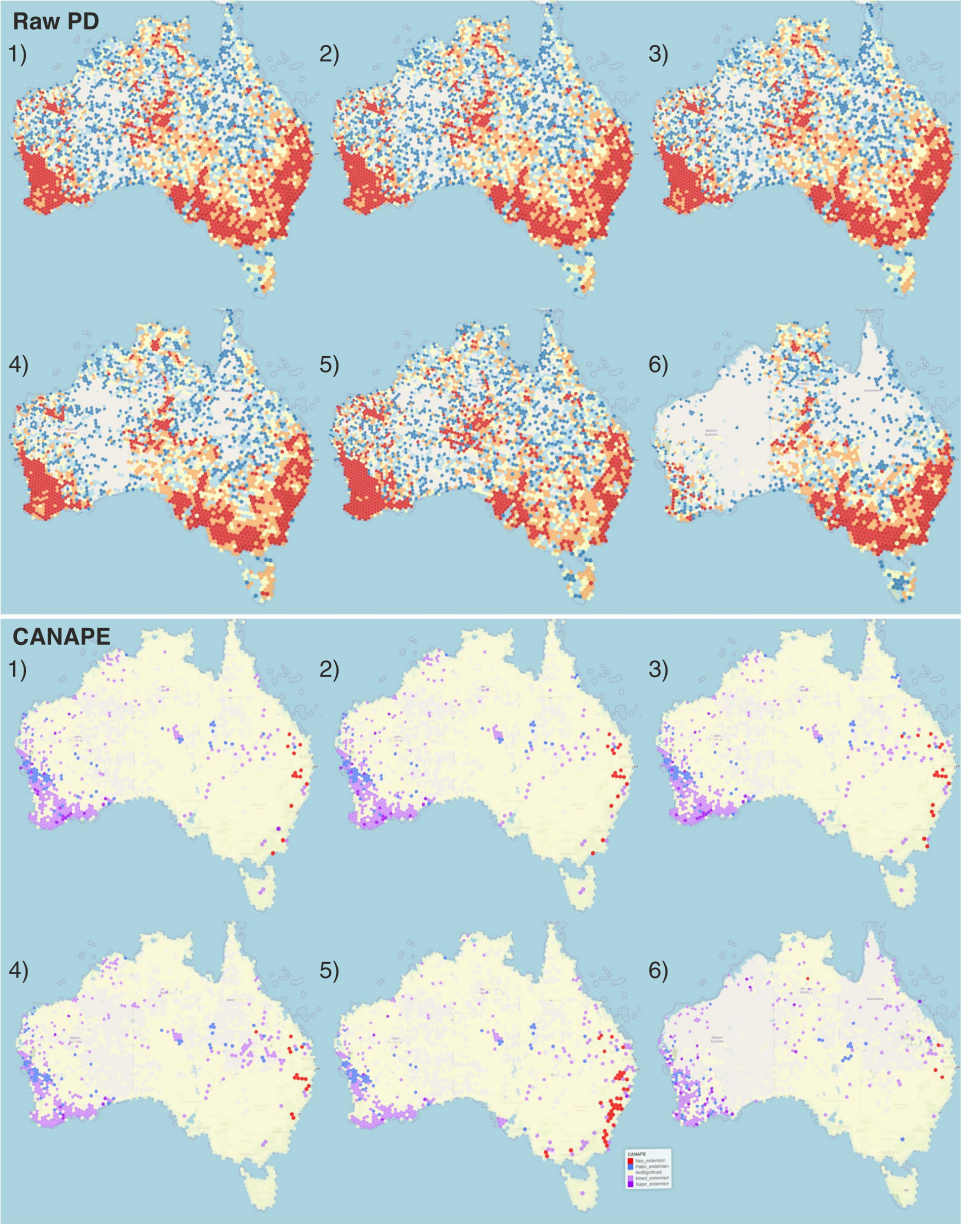
Effects of occurrence filtering and spatial outlier removal on the raw phylogenetic diversity and CANAPE analyses. 1) all relevant GBIF occurrences, 2) all relevant GBIF occurrences excluding geospatial errors (e.g., country centroids), 3) all relevant GBIF occurrences, excluding country centroids and outliers identified based on DBSCAN with stringent (500 km, 20 pts) and 4) low (50 km, 3 pts) detection thresholds, 5) the same settings as in 3, but only preserved specimen occurrences, and 6) the same settings as 3, but only observation occurrences

To evaluate the impact of excluding centroids and deleting putative outliers from occurrence data on the diversity estimates, we performed a second set of analyses using the original phylogeny and a range of GBIF occurrence data filtering settings (Fig. 4). To estimate standardized effect sizes of diversity measures, we performed 5000 randomization iterations.

The effect of the filtering is demonstrated with the increasing background in the raw PD maps (Fig. 4) indicating fewer grid cells with data points. The most stringent DBSCAN analysis has the fewest occupied grid cells. The results of this occurrence type analysis differ greatly, especially in Western Australia and Queensland due to the availability of observation data.

PhyloNext analysis of different subsets of GBIF data were similar in the CANAPE analysis that includes a spatial randomization. The restricted observation occurrence distributions likely affect the randomizations as well because the randomization only allocates taxa to spatial units with observations. Fewer grid cells have measured values that are identified as significantly different from random. During the randomization process there are fewer areas for a species to be distributed among, therefore the measured is more likely to be part of the normal distribution. This likely accounts for the lack of colored cells in NSW and the relative increase in Queensland (Fig. 4).

## Conclusion

The extensive and rich nature of GBIF data is indisputable, providing a wealth of valuable information for researchers and policymakers. However, the sheer volume and complexity of this data can prove daunting for new users, requiring specialized knowledge and experience to navigate it effectively. During data indexing, GBIF flags data for various quality attributes, which can be used to filter data downloads from the portal or via the API. To ensure correct data use citation, each download is assigned a DOI. However, filtering the downloaded data using various R packages such as *rgbif* and *CoordinateCleaner* [36] requires an experienced user which reduces the uptake of GBIF data use in non-research areas such as policy. Furthermore, obtaining a new DOI for the subset data is often neglected, thereby reducing the precision of data citation.

To address these challenges, PhyloNext bundles this workflow and provides access to common filters while providing an easy pathway to mint a DOI for citation for the exact dataset used via the derived dataset DOI tool (https://www.gbif.org/derived-dataset/about). The pipeline is a new workflow offering comprehensive and flexible tools for estimating phylogenetic diversity using GBIF-mediated species occurrence data and phylogenetic information from the Open Tree of Life. The custom synthesis pipeline available as part of the OpenTree project allows users to reproducibly and efficiently combine phylogenetic estimates from multiple published studies and infer smoothed dates for these synthetic trees. PhyloNext features spatial outlier detection, taxonomy name matching, diversity estimation, and visualization. The pipeline could be useful for identifying areas that are important for conservation, and to inform the research and development of conservation and biodiversity management strategies.

Our analyses indicate that data can be carefully filtered through the integrated pipeline and produce results nearly identical to highly curated datasets that take months of human curation time. The flexibility of PhyloNext enables analyses at any spatial or taxonomic level, significantly reducing the barrier of uptake for researchers. PhyloNext is designed as a transparent pipeline that records all intermediate data and analytical steps.

## Opportunities and future directions

PhyloNext has been developed to seamlessly integrate GBIF data and OpenTree phylogenies with the Biodiverse analytical engine, enabling the computation of key metrics like phylogenetic diversity, species richness, and endemicity. Its applicability across various taxa and geographies offers a broad spectrum of research possibilities, contingent upon the availability of adequate data. Certain changes in biodiversity, such as those caused by point polluters or pathogen outbreaks, may have specific spatial and temporal epicenters. The use of hierarchical indexing of H3 grid cells simplifies the identification of adjacent cells, facilitating the detection of the sources, directions, and, when coupled with temporal filters, the velocity of these ecological disturbances or changes.

The modular design of the PhyloNext pipeline allows for independent use or integration with other datasets and analyses. Given that GBIF-mediated data is versatile [1, 37], and the data needs to be fit for purpose, GBIF does not prescribe any specific data filters. Therefore, the responsibility for data selection and reuse is entrusted to the user, promoting a culture of careful and precise data management, particularly where the quality and appropriateness of data are crucial. While some researchers prefer this control, other user groups, such as policymakers or non-experts, could benefit from access to pre-filtered, community-validated datasets ready for immediate application.

GBIF-mediated data is widely used for developing species distribution models in evolutionary and conservation studies [1]. The PhyloNext data filtering module can be customized to supply modelers with specific pre-filtered data that meets their specific requirements. This feature could significantly enhance the speed, reproducibility, and citation of models, and expand modeling accessibility. A proposed initiative is the monthly generation of new datasets using a community-agreed set of filtering parameters. These datasets, each assigned a unique DOI for accurate citation, would be archived publicly with variable filter settings, thereby providing a valuable resource for a wide range of modeling requirements.

In many locations, the sparse nature of species occurrence records can lead to biases in diversity estimates. These biases stem from the assumption that the absence of data necessarily indicates species absence, which is not always true for under-studied areas. This problem could be addressed through species distribution modeling and subsequent diversity estimation based on predicted species ranges. The latest advancements in machine learning and artificial intelligence could be particularly useful, as they are capable of identifying complex patterns and relationships within sparse datasets that traditional methods might overlook. Our pipeline’s modular design allows the incorporation of these new techniques into the workflow.

In addition to the opportunities of cloud-based computing, GBIF has enabled an accessible interface to PhyloNext on GBIF.org, which will enable users to easily interact with and utilize the workflow. This user-friendly GUI will streamline the process of analyzing phylogenetic diversity metrics, making it more accessible to researchers and conservationists alike.

Phylogenetic diversity measures are incorporated into the monitoring framework of the Kunming-Montreal Global Biodiversity Framework (https://www.post-2020indicators.org/complementary-indicators). Metrics such as the EDGE index, which incorporates evolutionary distinctiveness similar to PD, serve as a component indicator; the Expected Loss of Phylogenetic diversity also acts as a complimentary indicator of Goal A. Additionally, the changing status of evolutionary distinct and globally endangered species, as measured by the EDGE index, is a complimentary indicator for Target 4.

GBIF is developing a roadmap for the implementation of PhyloNext in coordination with biodiversity monitoring programs. We anticipate the power of this pipeline will facilitate periodic analyses across major organismal groups, including angiosperms, gymnosperms, vertebrates, mammals, reptiles, butterflies, and fungi. These analyses are capable of calculating indices such as phylogenetic diversity at various grid resolutions, assessing overall global diversity, and examining diversity in specific regions like continents, countries, biomes, or other circumscribed areas. The resulting monthly data products, useful for indicators like EDGE and Essential Biodiversity Variables (EBVs), will offer a consistent and replicable framework for monitoring biodiversity changes at any scale. This approach will support the research and conservation planning community in addressing diverse questions, ranging from biogeography to the identification and protection of threatened or at-risk areas.

Lastly, the proposed workflow provides a strong foundation for developing educational resources and training programs aimed at empowering a wider audience to effectively utilize the pipeline. By offering accessible learning materials and hands-on training experiences, we can engage diverse stakeholders in conservation planning, policy development, and research initiatives. These educational efforts not only promote a broader understanding of phylogenetic diversity assessment techniques but also encourage collaborative problem-solving and informed decision-making in the pursuit of global biodiversity conservation goals.

## Data Availability

The original dataset (based on 10.5061/dryad.33kn3) in Parquet format and a subset of *Acacia* species occurrences deposited in GBIF (December 2022) along with phylogenetic trees and scripts accompanying the manuscript available at https://github.com/vmikk/PhyloNext_manuscript The pipeline's source code and documentation are available on GitHub and Zenodo: Pipeline documentation: https://phylonext.github.io/ Pipeline source code: https://github.com/vmikk/PhyloNext Source code archive at Zenodo: 10.5281/zenodo.7974081 (https://zenodo.org/record/7974081) Source code of the web GUI and the web service: https://github.com/gbif/phylonext-ui/, https://github.com/gbif/phylonext-ws All required dependencies, encapsulated in Docker and Singularity containers, are accessible on Docker Hub and the Singularity library: https://hub.docker.com/r/vmikk/biodiverse https://hub.docker.com/r/vmikk/rarrow https://hub.docker.com/r/vmikk/opentree https://cloud.sylabs.io/library/vmiks/gbif/biodiverse https://cloud.sylabs.io/library/vmiks/gbif/rarrow https://cloud.sylabs.io/library/vmiks/gbif/opentree Additionally, these containers are archived on Zenodo for long-term access 10.5281/zenodo.7973798 (https://zenodo.org/record/7973798). **Project name:** PhyloNext. **Project home page:** https://phylonext.github.io/ **Archived version:** 10.5281/zenodo.7974080 **Operating system(s):** Platform independent (Windows through WSL). **Programming languages:** R, Groovy, Perl, Python; JavaScript (Node.js) for the web-GUI. **Other requirements:** Internet connection (required for automatic fetching of phylogenetic trees and species name matching). Software dependencies for the standalone version of PhyloNext: Nextflow >= 22.10, Java >= 11, Docker >= 24.0 or Singularity >= 3.8. Requirements for the web-GUI: a commonly supported modern web browser (Google Chrome, Mozilla Firefox, Apple Safari, or Microsoft Edge) with JavaScript enabled, a user account at GBIF.org. **License:** MIT. **Any restrictions to use by non-academics:** None.

## Abbreviations

API: Application programming interface
CANAPE: Categorical analysis of neo- and paleoendemism
COL: Catalogue of Life
CWE: Corrected form of range-weighted endemism
DBSCAN: Density-based spatial clustering of applications with noise
DGGS: Discrete global grid system
DOI: Digital object identifier
EDGE: Evolutionarily distinct and globally endangered
EPSG: European petroleum survey group registry
GBIF: Global Biodiversity Information Facility
GIS: Geographic information system
GUI: Graphical user interface
H3: A geospatial indexing system developed by Uber
OSM: OpenStreetMap
OToL: Open Tree of Life
OTT: Open Tree Taxonomy
PD: Phylogenetic diversity
PE: Phylogenetic endemism
REST: Representational state transfer
RPD: Relative phylogenetic diversity
RPE: Relative phylogenetic endemism
SES: Standardized effect size
TD: Taxonomic diversity
TNRS: Taxonomic name resolution service
WE: Range-weighted endemism
WGS: World Geodetic System
